# Measuring ^129^Xe transfer across the blood‐brain barrier using MR spectroscopy

**DOI:** 10.1002/mrm.28646

**Published:** 2021-01-17

**Authors:** Madhwesha R. Rao, Graham Norquay, Neil J. Stewart, Jim M. Wild

**Affiliations:** ^1^ POLARIS, Department of Infection, Immunity and Cardiovascular Disease and Insigneo Institute of In‐silico Medicine University of Sheffield Sheffield UK

**Keywords:** blood‐brain barrier, gas‐exchange, hyperpolarized xenon‐129, time‐resolved magnetic resonance spectroscopy, tracer kinetic model

## Abstract

**Purpose:**

This study develops a tracer kinetic model of xenon uptake in the human brain to determine the transfer rate of inhaled hyperpolarized ^129^Xe from cerebral blood to gray matter that accounts for the effects of cerebral physiology, perfusion and magnetization dynamics. The ^129^Xe transfer rate is expressed using a tracer transfer coefficient, which estimates the quantity of *hyperpolarized* ^129^Xe dissolved in cerebral blood under exchange with *depolarized* ^129^Xe dissolved in gray matter under equilibrium of concentration.

**Theory and Methods:**

Time‐resolved MR spectra of hyperpolarized ^129^Xe dissolved in the human brain were acquired from three healthy volunteers. Acquired spectra were numerically fitted with five Lorentzian peaks in accordance with known ^129^Xe brain spectral peaks. The signal dynamics of spectral peaks for gray matter and red blood cells were quantified, and correction for the ^129^Xe *T*_1_ dependence upon blood oxygenation was applied. ^129^Xe transfer dynamics determined from the ratio of the peaks for gray matter and red blood cells was numerically fitted with the developed tracer kinetic model.

**Results:**

For all the acquired NMR spectra, the developed tracer kinetic model fitted the data with tracer transfer coefficients between 0.1 and 0.14.

**Conclusion:**

In this study, a tracer kinetic model was developed and validated that estimates the transfer rate of HP ^129^Xe from cerebral blood to gray matter in the human brain.

## 1 INTRODUCTION

**FIGURE 1:**
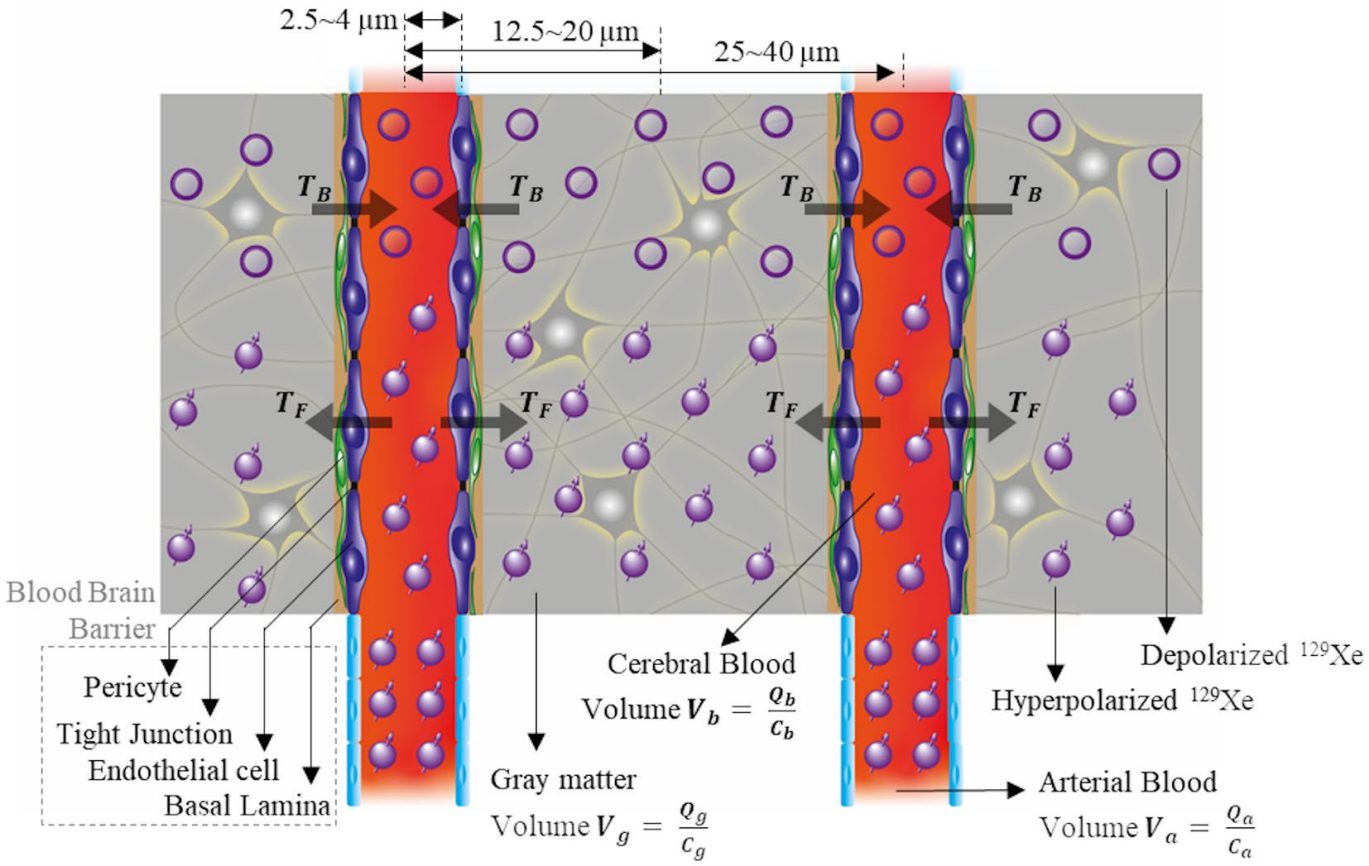
Illustration of BBB, cerebral blood volume for gray matter, gray matter volume, arterial blood volume and transfer of xenon across the barrier

The blood‐brain barrier (BBB) separates the intravascular space from the brain parenchyma, and is formed by specialized endothelial cells held together by tight junctions, covered by basal lamina and surrounded by pericytes, as shown in Figure 1. It acts as a selectively permeable barrier, which allows molecules essential for brain function through the barrier, while restricting the passage of noxious or neuroactive substances, and is vital for maintaining normal neuronal function.^1^ The consequence of a ruptured BBB is irregular transportation (and clearance) of gases, nutrients, water and other essential molecules across the barrier. These pathophysiological irregularities of an impaired BBB can be diagnostically assigned to various diseases processes,^2^, ^3^, ^4^, ^5^, ^6^ typically by observing the movement of a tracer across the barrier. However, dysfunction of an intact BBB can also occur due to prolonged oxidative stress, causing deposition of plaque (example amyloid β),^7^, ^8^, ^9^ build‐up of lipids, decline of pericytes,^10^ cognitive ability and brain function.^11^ This can occur even before the barrier becomes leaky or ruptured. For diagnosis, the two most commonly used tracers are iodinated contrast agent for X‐ray CT and gadolinium chelated contrast agent for dynamic contrast enhanced MRI,^12^, ^13^, ^14^, ^15^, ^16^ neither of which cross an intact BBB, but are able to when it is leaky or ruptured. The arterial spin labeling (ASL) based MRI technique^17^, ^18^, ^19^, ^20^, ^21^ images magnetically tagged water molecules in the arterial blood delivered to brain tissue. Since the water molecules cross the *intact* BBB, this technique is being investigated for diagnosis of BBB diseases.^22^, ^23^, ^24^ Nevertheless, the technique has some limitations due to acquisition strategy (post labeling delay time, labeling plane) competing with physiology (longitudinal relaxation time, arterial transit time) and abundance of water molecules in the brain tissue (background noise, magnetization transfer).^19^, ^25^

Xenon is an exogenous tracer that passively crosses the *intact* BBB.^1^ Recently, hyperpolarized (HP) xenon‐129 has been demonstrated as an agent to image the uptake of inhaled gas into human brain tissue after crossing the *intact* BBB^26^, ^27^ by MRI, and the method has been shown to be repeatable.^28^ Preliminary clinical investigations of HP ^129^Xe brain MRI have shown novel and complementary image contrast for stroke,^29^ Alzheimer’s disease^30^ and functional brain imaging.^31^ HP ^129^Xe dissolved in the brain exhibits discrete chemical shifts for various biochemical compartments such as cerebral red blood cells (RBC), cerebrospinal/interstitial fluid, gray matter, white matter and soft muscular fat.^27^ The image signal‐to‐noise ratio (SNR) and contrast is weighted by regional cerebral perfusion, physiology (tissue compartmental volumes, temperature, blood pressure) and gas‐transfer from cerebral blood to gray matter across the BBB,^26^ while the magnetization history of the HP ^129^Xe during its journey through these compartments is also factorial.

The transfer dynamics of HP ^129^Xe, a *passive* tracer, is not influenced by regional cellular metabolism, oxygen extraction^32^ or electrolytic balance. Thus, HP ^129^Xe MRI potentially holds unique information about the BBB by characterizing the *passive* transferability across the barrier. Nevertheless, this is influenced by the effects of regional brain physiology and perfusion. The HP aspect of the tracer adds further challenges as the MR detection is limited to the *HP* pool of ^129^Xe,^33^ but the gas transfer (membrane diffusion) dynamics are influenced by the concentration of all ^129^Xe atoms in the respective compartments, some of which may not be HP and therefore are undetectable.

This study develops a tracer kinetic model to determine the transfer rate of HP ^129^Xe from the cerebral blood to gray matter by considering the mass transfer of ^129^Xe irrespective of its status of hyperpolarization, with the aim of quantitatively measuring the compartmental transfer dynamics in order to characterize the BBB function. In contrast to earlier mathematical modeling studies of ^129^Xe uptake in brain tissue that arrived at the concentration of ^129^Xe over time duration,^34^, ^35^, ^36^ the model presented here uses the concentrations of ^129^Xe in cerebral blood and gray matter compartments measured using NMR spectroscopy to estimate the transfer dynamics between them. The proposed model also considers the variation in longitudinal relaxation of HP ^129^Xe dissolved in the blood by monitoring the variation in oxygenation‐dependent chemical shift of RBC spectral peak.

## 2 THEORY

### 2.1 Tracer kinetic model for HP ^129^Xe uptake in the brain

After inhalation of HP ^129^Xe, it crosses the alveolar‐capillary barrier and is transported to the brain through the systemic circulation in approximately 4 s.^37^ In the brain, ^129^Xe crosses the intact BBB passively in to the brain parenchyma,^1^ and thereafter is under continuous diffusional exchange across the barrier. The cerebral blood volume of ~4 mL for 100 g of gray matter^37^, ^38^, ^39^ and cerebral capillary of radius 2.5~4 µm^40^, ^41^, ^42^, ^43^ implies that the radius of the gray matter coaxial with the capillary and mean distance between capillaries would be 12.5~20 µm and 25~40 µm^40^, ^41^, ^42^, ^43^ respectively, as shown in Figure 1. As the diffusion coefficient of xenon dissolved in the cerebral blood or gray matter has not been characterized, it can be approximated by using the diffusion coefficient dissolved in water of ~1 × 10^−5^ cm^2 ^s^−1^.^44^ Under these conditions, a 95% equilibrium of concentration of ^129^Xe between cerebral blood and gray matter is reached in 0.2~0.4 seconds.^45^ Hence, the NMR spectral peaks in time‐resolved spectra that are acquired using a sequence repetition time (TR), that is longer than both the *cerebral mean transit time* (≈3.3 s^39^, ^46^, ^47^) and *equilibrium time* (0.4 s), are linearly proportional to the concentration in the respective biochemical compartments.

Consider time‐resolved NMR spectroscopic acquisitions where each acquisition consists of a RF excitation pulse and subsequent saturation pulses. After every acquisition the bulk magnetization of HP ^129^Xe is *depolarized* (DP) to thermal equilibrium such that the NMR signal from further excitation of this pool of ^129^Xe in the brain is too weak to be detectable. Therefore, in each of the acquired time‐resolved spectra, the spectral peaks do not represent the total quantity; instead, they represent the quantity of *HP* ^129^Xe accumulated between two consecutive NMR acquisitions. Nevertheless, there is continuous accumulation of DP ^129^Xe in the brain. Due to their respective discrete chemical shifts,^27^ the change in the relative concentration of HP ^129^Xe in cerebral blood and gray matter can be monitored over time, and thus HP ^129^Xe can be used as a tracer to examine the transfer dynamics across the shared barrier. Because there is a continuous arterial in‐flow of HP ^129^Xe to the cerebral vasculature, the *forward transfer* (*T_F_*) of ^129^Xe from cerebral blood to gray matter is HP ^129^Xe and therefore is detectable, annotated as *T_F_* in Figure 1. We define the *T_F_* rate as the quantity of HP ^129^Xe delivered to gray matter in the time duration (TR) between two successive acquisitions, considering both arterial in‐flow of HP ^129^Xe and diffusional gas‐exchange across the BBB. Due to the radiofrequency (RF) saturation pulses used after each acquisition, the *backward transfer* of ^129^Xe from gray matter to cerebral blood is DP ^129^Xe and therefore is undetectable, annotated as *T_B_* in Figure 1. NMR detection depends on the magnetization (*M*), which is proportional to the quantity (*Q*) and polarization of ^129^Xe, whereas the transfer dynamics depends on concentration (*C*), which is proportional to the quantity irrespective of its status of polarization.

For a given cerebral blood flow for gray matter *F*, partition coefficient of xenon between gray matter and blood *λ*, cerebral mean transit time *ψ*, cerebral blood volume *V_Blood_* per volume of gray matter *V_GM_*, concentration of HP ^129^Xe in arterial blood *C_A_* and NMR spectral repetition time *t_TR_*, using Fick’s principle developed for an inert gas tracer by Kety,^45^ we can arrive at the concentration of ^129^Xe in gray matter *C_GM_* for the time instance immediately after the (*n*−1)^th^ RF acquisition as shown in Equation (1); nevertheless, this pool of ^129^Xe would be DP ^129^Xe.

(1) $$C_{GM,DP}\;=\;\lambda C_{A}\left(1‐e^{‐\frac{t_{\mathrm{TR}}\left(n‐1\right)}{V\psi }}\right)$$

where, $F\lambda^{‐1}V_{\mathrm{GM}}^{‐1}=V_{\mathrm{Blood}}\lambda^{‐1}V_{\mathrm{GM}}^{‐1}\Psi^{‐1}=V^{‐1}\Psi^{‐1}$ and *V* ^−1^ = *V_Blood_* *λ* ^−1^ $V_{\mathrm{GM}}^{‐1}$. The quantities of HP (*Q_GM_*_,_ *_HP_*) and DP (*Q_GM_*_,_ *_DP_*) ^129^Xe in the gray matter at the time instance at the *n* ^th^ RF acquisition are:

(2) $$Q_{GM,HP}\;=\;\lambda V_{\mathrm{GM}}C_{A}\left(1‐e^{‐\frac{t_{\mathrm{TR}}}{V\psi }}\right)$$

$$Q_{GM,DP}\;=\;\lambda V_{\mathrm{GM}}C_{A}\left(1‐e^{‐\frac{t_{\mathrm{TR}}\left(n‐1\right)}{V\psi }}\right)$$

Such that, the total quantity of xenon is $Q_{\mathrm{GM}}=Q_{GM,HP}+Q_{GM,DP}$. Between the time instances of the ${\left(n‐1\right)}^{\mathrm{th}}$ and $n^{\mathrm{th}}$ RF acquisition, there is continuous diffusional exchange of ^129^Xe between gray matter and cerebral blood. If we let r be the fraction of ^129^Xe in gray matter that would exchange with ^129^Xe in cerebral blood, we have $Q_{\mathrm{GM}}=\left(1‐r\right)Q_{\mathrm{GM}}+rQ_{\mathrm{GM}}$, where $rQ_{\mathrm{GM}}$ is the quantity of ^129^Xe that would exchange with cerebral blood. Further, because we have two pools of ^129^Xe (HP and DP) in both gray matter and cerebral blood, for the fraction of ^129^Xe that would exchange with cerebral blood, we have four possibilities of exchange: (i) HP ^129^Xe being replaced by HP ^129^Xe $Q_{GM,HP↔HP}$, (ii) HP ^129^Xe being replaced by DP ^129^Xe $Q_{GM,HP↔DP}$, (iii) DP ^129^Xe being replaced by DP ^129^Xe $Q_{GM,DP↔DP}$, and (iv) DP ^129^Xe being replaced by HP ^129^Xe $Q_{GM,DP↔HP}$. Letting $k_{1}$ and $k_{2}$ be the factors that determine the fraction of ^129^Xe of a particular pool being replaced by the same pool, we can then derive the six‐term expression;

(3) $$Q_{\mathrm{GM}}\;=\;\left(1‐r\right)\;\left(Q_{GM,HP}\;+\;Q_{GM,DP}\right)\;+\;r\left(k_{1}Q_{GM,HP↔HP}+\;\left(1‐k_{1}\right)Q_{GM,HP↔DP}\;+k_{2}Q_{GM,DP↔DP}\;+\;\left(1‐k_{2}\right)Q_{GM,DP↔HP}\right)\;$$

The factors ${(1‐k}_{1})$ and $\left(1‐k_{2}\right)$ are the tracer transfer constants that determine the fraction of one pool of ^129^Xe being replaced by the other pool. For simplicity of calculation in the current context, we use; ${k=k_{1}=k}_{2}$. In Equation (3), only the first, third and last term relate to HP ^129^Xe which contributes to the NMR signal, thus rewriting and substituting from Equation (2), we have;

(4) $$Q_{G\mathrm{M,}\;HP}\;=\;\lambda V_{\mathrm{GM}}C_{A}\left[(1‐r(1‐k))\left(1‐e^{‐\frac{t_{\mathrm{TR}}}{V\psi }}\right)\;+\;r(1‐k)\left(1‐e^{‐\frac{t_{TR\;}(n‐1)}{V\psi }}\right)\right]$$

Equation (4) assumes that the gray matter compartment is solely in exchange with the cerebral blood compartment, and vice versa. The factor r that determines the fraction of ^129^Xe that would exchange with cerebral blood depends on several factors such as the capillary diameter, the mean distance between capillaries and the diffusivity of ^129^Xe. Consider the diffusivity of ^129^Xe in water *D* = 1 × 10^3^ µm^2^s^−1^ and the mean diffusive displacement from gray matter volume to cerebral blood volume *d_GM_*_→_ *_Blood_* ≈ 12.5 ~ 20 µm from Figure 1,^40^, ^41^, ^42^, ^43^ the time taken $\left(t_{GM\to Blood}\right)$ for diffusion from gray matter to cerebral blood volume is approximately;

$$t_{GM\to Blood}\;=\;\frac{1}{2}d_{GM\to Blood}^{2}D^{‐1}\approx 75∼200\;ms$$

Thus, the factor r can be approximated as;

$$r=\left\{\begin{array}{c} 1\\ \left(t_{\mathrm{TR}}‐\psi \right)\\ 0 \end{array}\right)t_{GM\to Blood}^{‐1}\;\;\;\;\;\;\;\;\begin{array}{c} \forall \left(t_{\mathrm{TR}}‐\psi \right)t_{GM\to Blood}^{‐1}>1\\ \forall \;\;1>\left(t_{\mathrm{TR}}‐\psi \right)t_{GM\to Blood}^{‐1}>0\\ \forall \;0>\left(t_{\mathrm{TR}}‐\psi \right)t_{GM\to Blood}^{‐1} \end{array}$$

For the quantity of HP ^129^Xe in cerebral blood, $Q_{Blood,HP}$, we have $\frac{dC_{Blood,HP}}{\mathrm{dt}}=\frac{F}{\lambda V_{\mathrm{GM}}}C_{A}$, where $C_{Blood,HP}$ is the concentration of HP ^129^Xe in cerebral blood. Rearranging and integrating for the time interval $t_{\mathrm{TR}}$, we have; $C_{Blood,HP}=\frac{t_{\mathrm{TR}}}{V\Psi }C_{A}$, and $Q_{Blood,HP}$ as;

(5) $$Q_{Blood,HP}=\;\frac{t_{\mathrm{TR}}}{V\psi }V_{\mathrm{Blood}}C_{A}$$

Similarly, for the quantity of HP ^129^Xe in arterial blood $Q_{A,HP}$, $Q_{A,HP}=C_{A}V_{A}$, where $V_{A}$ is the arterial blood volume between the lungs and the brain.

To corroborate tracer kinetics with the detected NMR spectroscopy, let $M_{0}$ be the bulk magnetization, such that $M_{0}\approx QP\Phi$, where Q is the quantity of ^129^Xe and Φ depends on gyromagnetic ratio, reduced Planck’s constant and nuclear spin quantum number.^48^ P is the polarization of the sample, such that $P_{\mathrm{HP}}$ and $P_{\mathrm{DP}}$ are the polarization of the *HP* pool of ^129^Xe that is detectable and DP pool of ^129^Xe that is not detectable respectively. The magnetization in transverse plane is propositional to $M_{0}$, weighted by flip angle and transverse relaxation time $\left(T_{2}^{*}\right)$. For a 90° flip angle, the acquired NMR signal is given by $M_{0}e^{j{2\pi f}_{0}t}e^{‐\frac{t}{T_{2}^{*}}}$, where $f_{0}$ is the center frequency of the spectral peak. The corresponding Fourier transform is given by $M_{0}{\left({\left(T_{*}^{2}\right)}^{‐1}+j2\pi (f_{0}‐f)\right)}^{‐1}$,^49^ which is a Lorentzian function $L\left\{\delta ,f\right\}$ if we let $T_{2}^{*}={\left(\pi \delta \right)}^{‐1}$, where δ is the half power peak width. Further, integrating the spectrum, we have $M_{0}\int_{‐\infty }^{\infty }L\left\{\delta ,f\right\}=M_{0}$, and thus, the magnitude of the spectral peak ($M_{\mathrm{GM}},M_{\mathrm{Blood}}$) measures the quantity ($Q_{\mathrm{GM}},Q_{\mathrm{Blood}}$) of ^129^Xe for a given polarization. $P_{\mathrm{DP}}$ depends on the strength of the static magnetic field, Boltzmann’s constant, gyromagnetic ratio, reduced Planck’s constant and temperature of ^129^Xe.^48^ $P_{\mathrm{HP}}$ depends on the polarization achieved by the spin‐exchange optical pumping process and $P_{\mathrm{HP}}\approx P_{\mathrm{DP}}10^{5}$.^33^ The polarization $\left(P_{\mathrm{HP}}\right)$ decays by longitudinal relaxation in several distinct biochemical compartments on its journey to the brain; in the gas‐phase in the lungs $T_{1_{\mathrm{GAS}}}$, in the dissolved‐phase in the blood $T_{1_{Blood,L\to B}}$ during the lung‐to‐brain (L→B) transit time $\tau_{L\to B}$ and in the dissolved‐phase in the gray matter $T_{1_{\mathrm{GM}}}$ during the TR $\left(t_{\mathrm{TR}}\right)$. Considering the decay of polarization, for a detectable NMR spectral peak at the $n^{\mathrm{th}}$ acquisition, we have the longitudinal magnetization as:

(6) $$M_{A}\;=\;\Phi P_{\mathrm{HP}}\Gamma Q_{\mathrm{A,}\;HP}e^{‐\frac{t_{TR}n}{T_{1_{GAS}\;}}}e^{‐\frac{\tau_{L\to B}}{T_{1_{Bloo\mathrm{d,}\;L\to B}}}}$$

$$M_{\mathrm{Blood}}\;=\;\Phi P_{\mathrm{HP}}b_{\mathrm{RBC}}Q_{Bloo\mathrm{d,}\;HP}e^{‐\frac{t_{\mathrm{TR}}}{T_{1_{Blood}}}}$$

$$M_{\mathrm{GM}}\;=\;\Phi P_{\mathrm{HP}}Q_{G\mathrm{M,}\;HP}e^{‐\frac{t_{\mathrm{TR}}}{T_{1_{GM}}}}$$

where, $b_{\mathrm{RBC}}$ is a scalar factor determining the quantity of ^129^Xe in the RBCs in the *whole* of the head when compared to the total quantity of ^129^Xe in cerebral blood that is in exchange with gray matter. Γ is a factor defining the dynamics of ^129^Xe signal in the lungs, such as exchange rate across the alveolar‐capillary barrier and the relative volumes/pressure of alveoli and pulmonary capillaries. Considering the ratio of $M_{\mathrm{GM}}$ by $M_{\mathrm{Blood}}$ and rearranging the terms, we have:

(7) $$\frac{M_{\mathrm{GM}}}{M_{\mathrm{Blood}}}=\;\frac{Q_{G\mathrm{M,}\;HP}}{e^{‐\frac{t_{\mathrm{TR}}}{T_{1_{Blood}}}}}\;\left(\frac{e^{‐\frac{t_{\mathrm{TR}}}{T_{1_{GM}}}}}{b_{\mathrm{RBC}}Q_{Bloo\mathrm{d,}\;HP}}\right)$$

Equation (7) is the tracer kinetic model for transfer dynamics of *HP* ^129^Xe in the brain and is the estimate of the *forward transfer* $T_{F}$ rate in Figure 1.The transfer dynamic is independent of $Q_{A,NMR}$ and the term in the brackets in Equation (7) is a constant. Although $‐\frac{t_{\mathrm{TR}}}{T_{1_{\mathrm{Blood}}}}$ is independent of time, $T_{1_{\mathrm{Blood}}}$ varies with the oxygenation of cerebral blood. Nevertheless, this dependence of $T_{1_{\mathrm{Blood}}}$ is well established in the literature and the chemical shift of the RBC spectral peak can be used to monitor variations in blood oxygenation and apply a *T_1_* correction accordingly.^32^, ^50^, ^51^, ^52^ Table 1 provides a list of the various parameters used in the study.

**TABLE 1:** List of key parameters used for the tracer kinetic model and analysis

Parameter	Symbol	Value	Reference
Cerebral blood volume for 100 g of gray matter *V_GM_*	*V*_Blood_	~4 mL	37, 38, 39
Radius of cerebral capillaries	‒	2.5 ~ 4 µm	40, 41, 42, 43
Mean distance between capillaries	‒	25 ~ 40 µm	40, 41, 42, 43
Diffusion coefficient of ^129^Xe dissolved in water	*D*	1 × 10^3^µm^2^ s^−1^	^44^
Time to reach 95% equilibrium in concentration	‒	0.2 ~ 0.4 s	^45^
^129^Xe Partition coefficient gray matter/blood	*λ*	0.88	^56^
Cerebral mean transit time	*ψ*	3.3 s	39, 46, 47
Longitudinal relaxation of ^129^Xe in blood	T_1_ *_Blood_*	4.5 s	32, 50, 51, 52
Transverse relaxation of ^129^Xe in blood	‒	2 ms	^54^
Transverse relaxation of ^129^Xe in gray matter	‒	8.8 ms	^27^
Factor *r*	*r*	1	This study
^129^Xe displacement time	*t_GM_*_→_ *_Blood_*	75 ~ 200 ms	This study
Repetition Time (TR)	*t_TR_*	4 s	This study

## 3 METHODS

In vivo MR brain spectroscopy with ^129^Xe was performed with approval from the UK National Research Ethics Committee. Spectroscopic experiments were conducted on three healthy male volunteers aged 25 y, 33 y and 34 y, and repeated three times for each of the volunteers. The heart rate and SO_2_ were monitored throughout the breath hold, which lasted no more than 24 s.

Experiments were performed on a GE HDx 1.5 T clinical MRI scanner. ^129^Xe gas was HP to $P_{\mathrm{HP}}>30\%$ polarization using a POLARIS (Sheffield, UK) regulatory approved spin exchange optical pumping polarizer.^53^ HP ^129^Xe gas of 500 mL was mixed with $N_{2}$ for a total inhaled dose of 1 L. An eight‐leg band‐pass birdcage RF coil was used^27^ for transceiver; with inner diameter of 300 mm and length of 295 mm. The gas mixture was administered by inhalation from a Tedlar bag as described in Rao et al.^26^ Time‐resolved whole‐brain spectra were acquired during a breath‐hold, so that the HP ^129^Xe gas mixture was maintained as a reservoir in the lungs supplying the dissolved ^129^Xe plasma and RBC signal to the brain through alveolar‐capillary gas exchange and onward through the systemic circulation.

MR spectroscopy parameters were as follows: center frequency = 17660800 Hz (198 ppm downfield from the ^129^Xe gas phase resonance), flip angle = 90°, non‐selective RF hard‐pulse with duration of 500 µs, receiver bandwidth = 1.2 kHz and number of sample points = 128. The acquisition time was ~107 ms, much longer than $T_{2}^{*}$ of ^129^Xe in RBC (2 ms^54^) and gray matter (8.8 ms^27^). TR was set to 4 s, which is longer than the typical cerebral mean transit time (Ψ) of 3.3 s^39^, ^46^, ^47^ and the equilibrium time of 0.4 s,^45^ assuming Ψ will not increase by more than 20% during the breath‐hold.^55^ Acquisition of time‐resolved spectra was initiated immediately after the inhalation of the gas dose with two cycles of non‐selective 90° RF pulses to destroy the polarization of the initial unknown quantity of HP ^129^Xe in the brain. After each subsequent pulse‐acquire NMR spectroscopic acquisition, two non‐selective 90° RF pulses were applied to destroy the polarization of the residual HP ^129^Xe.

A summation of five complex Lorentzian peaks in accordance with the known five spectral peaks of ^129^Xe dissolved in the human head^27^, ^36^ was numerically fitted to the acquired NMR spectra, and each of the spectral peaks were quantified as a product of π, height and width of the peak. These quantified spectral peaks represent the bulk magnetization of HP ^129^Xe in corresponding compartments, namely $M_{\mathrm{GM}}$ and $M_{\mathrm{Blood}}$ for gray matter and cerebral RBC compartments, respectively.

The transfer dynamics of HP ^129^Xe between gray matter and cerebral blood was determined by evaluating the ratio of the time course of the spectral peaks of ^129^Xe in gray matter and cerebral RBC $\left(M_{\mathrm{GM}}M_{\mathrm{Blood}}^{‐1}\right)$. Using typical physiological values; cerebral blood volume for gray matter of 4 mL per 100 g of tissue,^37^, ^38^, ^39^ cerebral mean transit time of 3.3 s^39^, ^46^, ^47^ and the partition coefficient for xenon dissolved in gray matter to blood as 0.88^56^ (see Table 1); the tracer kinetic model in Equation (7) was rescaled and numerically fitted to the acquired transfer dynamics using a range of tracer transfer constants (1‐k)=0 to 1. The error in the numerical fit was calculated as the standard deviation of the difference between the tracer kinetic model from Equation (7) and the acquired transfer dynamic ratio, expressed as a percentage by normalizing by the mean value of the acquired transfer dynamic ratio.

## 4 RESULTS

**FIGURE 2:**
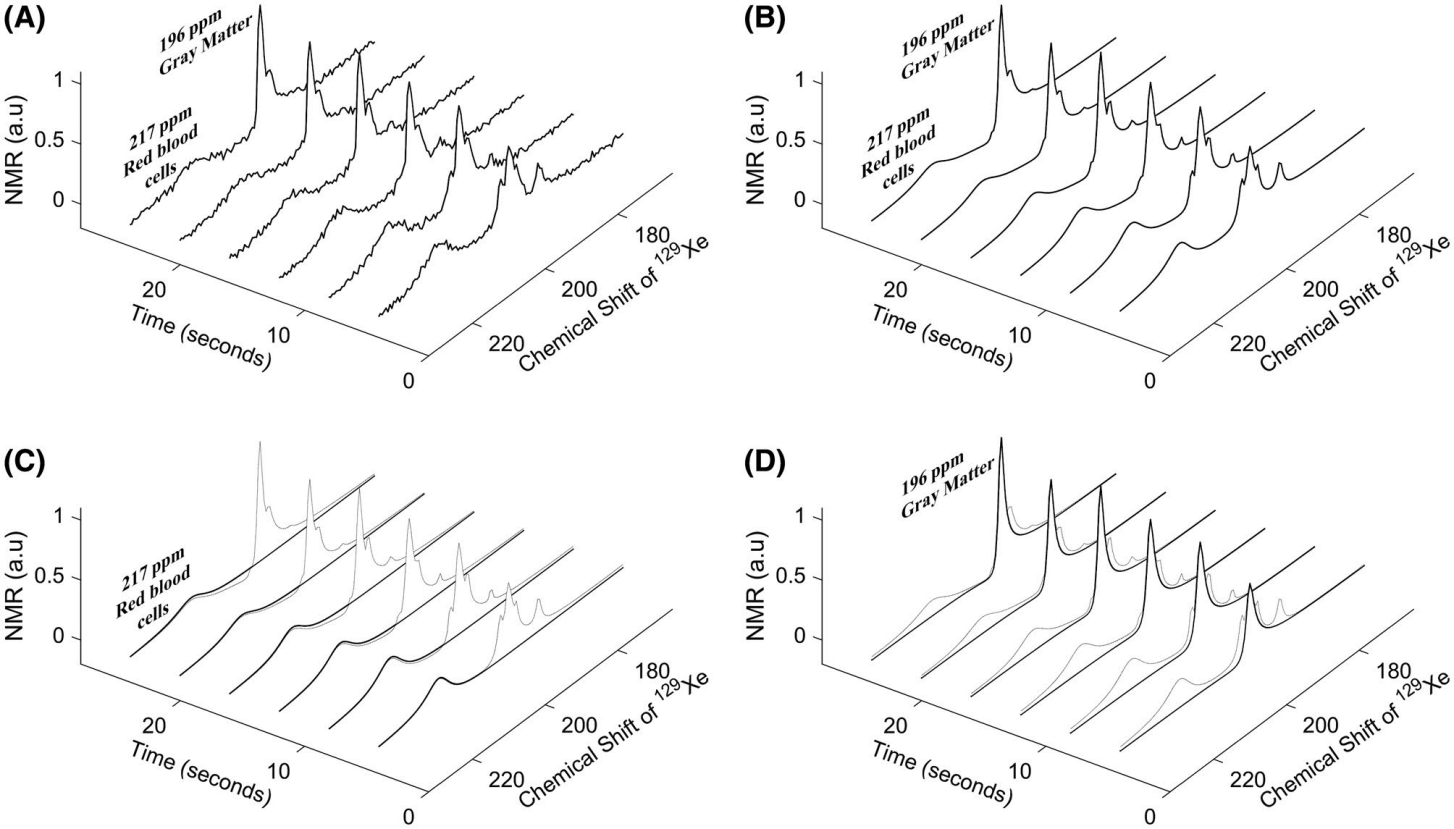
A, Acquired spectra for volunteer 34 y old, male. B, Artificially generated spectra comprising five complex Lorentzian peaks to numerically fit the acquired spectra in (A). Artificially generated complex Lorentzian peak for cerebral red blood cells (C) and gray matter (D)

A typical series of time‐resolved spectra acquired from the head of the 34‐y‐old male volunteer is shown in Figure 2A. Summation of five complex Lorentzian peaks fitted to the acquired spectrum in Figure 2A is shown in Figure 2B. The time course of the individual spectral peaks of HP ^129^Xe dissolved in cerebral RBC and gray matter is shown in Figure 2C and D, respectively.

**FIGURE 3:**
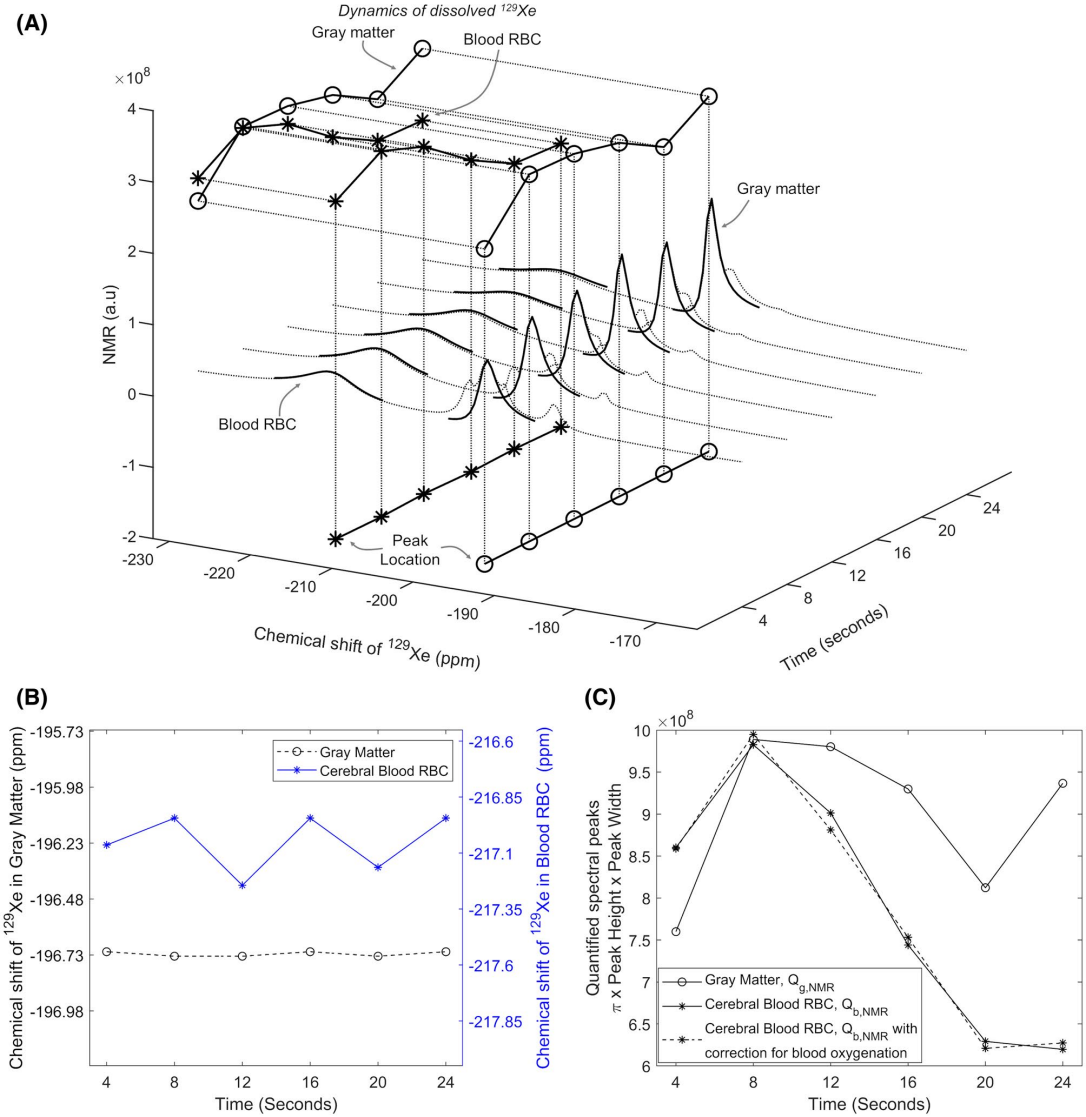
A, Illustration of the quantification and time course of spectral peaks for red blood cells and gray matter. Projection on *Time‐chemical shift* plane – time course of the peak location of both spectral peaks. Projection on *Time‐NMR unit* plane – time course of both quantified spectral peaks. *Note: although peaks were quantified as the product of π, height and width of the peak, only the product of height and width of the peak is shown here for ease of illustration*. B, Variation in chemical shift for both peaks; cerebral red blood cells and gray matter. C, Time course of spectral peaks from gray matter and cerebral red blood cells, with and without correction of the RBC peak for variation in *T*_1_ due to blood oxygenation, derived using the variation in chemical shift in (B)

Quantification of the spectral peaks for cerebral blood and gray matter is shown in Figure 3A along with the time course of the individual quantified spectral peaks. The chemical shifts of both individual spectral peaks over the time‐course of the acquisition are shown in Figure 3B. Variation in chemical shift can be observed for the cerebral RBC peak. This indicates underlying variations in the oxygenation of cerebral blood and is used to determine the variation in $T_{1}$ associated with it. The time courses of both quantified individual spectral peaks are shown in Figure 3C. Figure 3C also shows the correction to the quantification of the cerebral RBC peak for variation in $T_{1}$ of ^129^Xe in the RBCs derived using the variation in chemical shift from Figure 3B.

**FIGURE 4:**
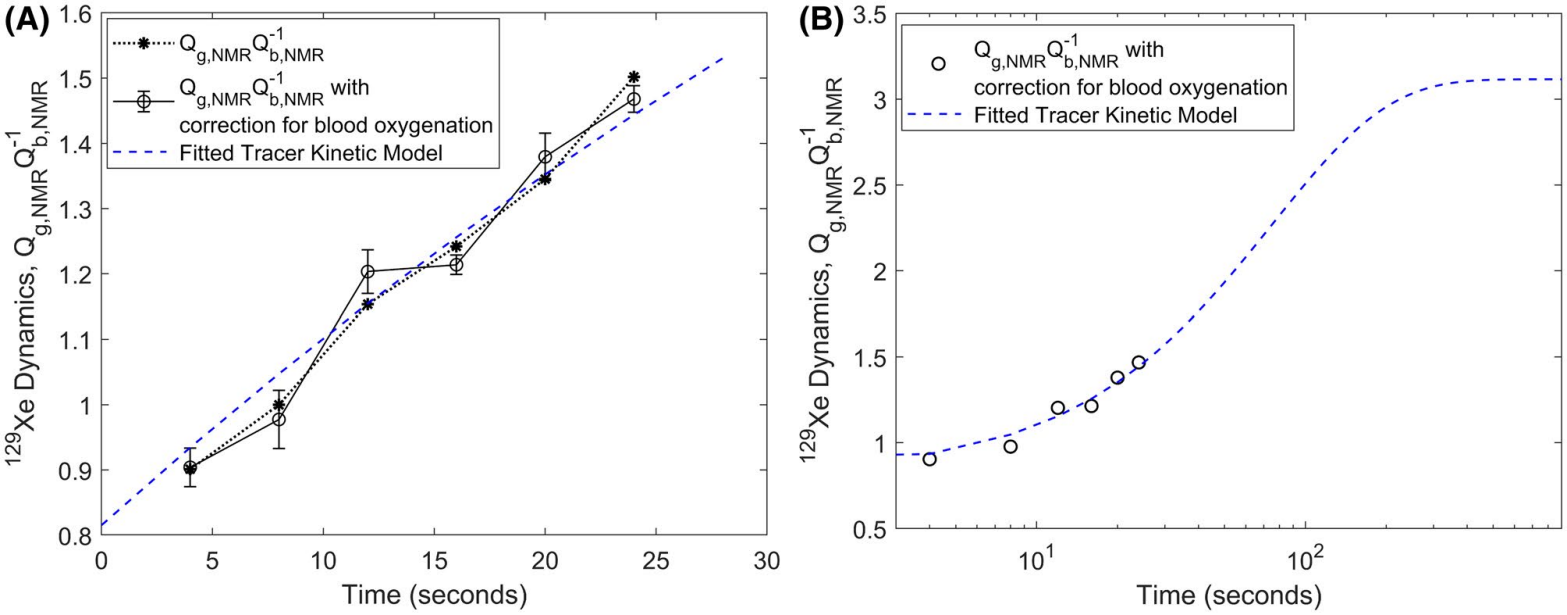
A, Transfer dynamics of ^129^Xe between cerebral blood and gray matter, with and without correction of the RBC peak (see Figure 3C), along with a rescaled and fitted tracer kinetic model (from Equation 7) with a tracer transfer constant (1−*k*) = 0.12. Error bars calculated considering three repetitions. B, Extrapolation of the tracer kinetic model to illustrate that the *forward transfer* (*T_F_*) rate of HP ^129^Xe reaches 95% saturation at approximately 200 s

**FIGURE 5:**
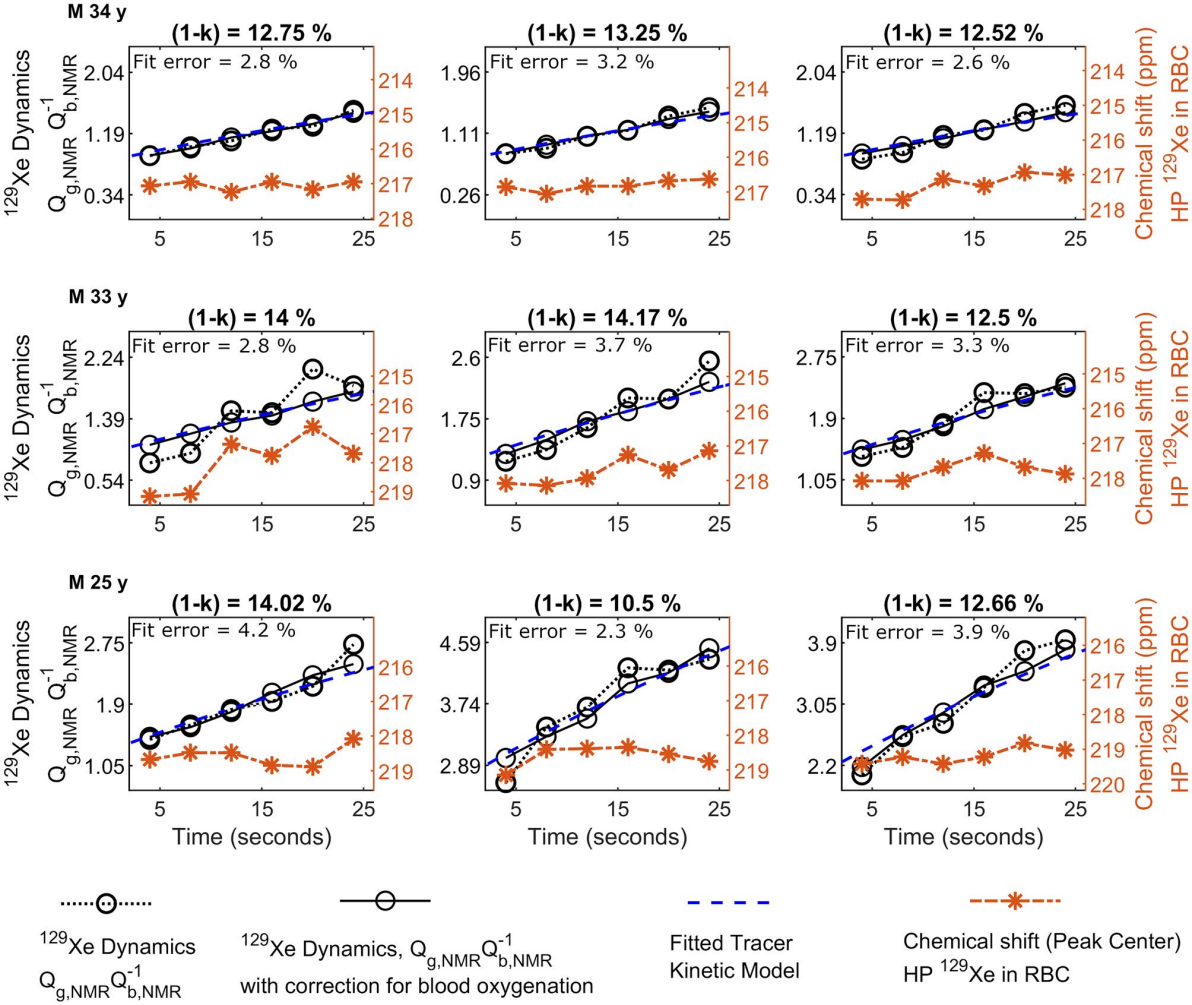
Transfer dynamics with and without correction due to variation in *T*_1_ of red blood cells, along with the rescaled and fitted tracer kinetic model from Equation (7), for three volunteers (rows) and for three separate repetitions (columns). For each of the acquisitions, the variation in chemical shift of ^129^Xe in red blood cells is shown. Error in the numerical fit is also indicated

The transfer dynamics of HP ^129^Xe between gray matter and cerebral blood is shown in Figure 4A, both with and without the correction for cerebral blood oxygenation applied to the RBC peak, along with the rescaled and fitted tracer kinetic model from Equation (7) for the tracer transfer constant (1‐k)=0.12. Extrapolation of the model to the case of steady‐state xenon tissue saturation is shown in Figure 4B, and indicates that the *forward transfer* ($T_{F}$) rate of HP ^129^Xe reaches 95% saturation after approximately 200 s. The transfer dynamics of HP ^129^Xe between gray matter and cerebral blood for all the three volunteers for all the three repeats are shown in Figure 5.

For each of the acquisitions, the transfer dynamics with and without the correction for blood oxygenation, along with the variation in chemical shift of ^129^Xe in RBCs and the numerical fit of the tracer kinetic model in Equation (7) are shown. The developed tracer kinetic model returned values for the tracer transfer constant between 0.1 and 0.14 over the three volunteers. The error in the numerical fit was between 2.3% and 4.2% as indicated in Figure 5.

## 5 DISCUSSION

The unique aspects of the proposed MR spectroscopy technique based on a tracer kinetic model are as follows; First, the dynamics of the relative quantity of ^129^Xe is quantified using spectral peaks separated by their distinct chemical shifts, avoiding potential uncertainties related to non‐specific binding and partial volume estimates for gray matter and blood which appear to vary with disease progression and aging in the brain.^13^, ^57^, ^58^, ^59^, ^60^ Second, the NMR signal from the tracer (HP ^129^Xe) once detected after RF excitation becomes NMR invisible (*DP*), enabling the observation of the physiological dynamics of cerebral uptake and diffusional exchange.

An interesting observation of the model is that it predicts that the *forward transfer* ($T_{F}$) rate ($M_{\mathrm{GM}}M_{\mathrm{Blood}}^{‐1}$ from Equation 7) increases until saturation. This is because, over time, *DP* ^129^Xe accumulates in the gray matter that will diffuse back (*backward transfer* $T_{B}$) in to cerebral blood and displace an equal amount of *HP* ^129^Xe (*forward transfer* $T_{F}$) from cerebral blood to gray matter. This exchange occurs without disrupting the equilibrium in concentration of overall ^129^Xe (irrespective of its status of polarization) between the two compartments. In addition, due to continuous arterial blood flow carrying *fresh* HP ^129^Xe to the head, the concentration of DP ^129^Xe in cerebral blood is much lower than that of gray matter increasing the likelihood of DP ^129^Xe in gray matter being replaced by HP ^129^Xe. Extrapolation of the model to 200 s and beyond indicates that the model ${(M}_{\mathrm{GM}}M_{\mathrm{Blood}}^{‐1}$) quickly saturates and is $\lambda V_{\mathrm{GM}}C_{A}b_{\mathrm{RBC}}^{‐1}Q_{Blood,HP}^{‐1}e^{‐\frac{t_{\mathrm{TR}}}{T_{1_{\mathrm{GM}}}}}e^{\frac{t_{\mathrm{TR}}}{T_{1_{\mathrm{Blood}}}}}\left(1‐ke^{‐\frac{t_{\mathrm{TR}}}{V\Psi }}\right)$ at acquisition n=∞. Nevertheless, this saturation time does not have physiological interpretation in the current context.

The factors $k_{1}$ and $k_{2}$ may not be equal, and estimating these factors individually and their inter‐relationship is the scope of future study. The key limitations of the model are that the spectral measurements are performed over the whole brain, and thus average values are considered for cerebral blood flow, cerebral blood volume, mean transit time and gray matter volume. Using spatially resolved spectroscopy and for a known *regional* mean transit time and cerebral blood volume for gray matter, that can be estimated from ^1^H MRI for example,^61^ the proposed tracer kinetic model estimates the *regional* efficacy of ^129^Xe transfer or uptake across the intact BBB. This might provide additional useful insight in to the underlying *regional* pathophysiology, such as BBB surface area and *intact‐barrier* permeability/transferability changes. An in depth clinical investigation of the method in this aspect is the scope of future work. Also, if the mean transit time is known *a priori*, the NMR pulse repetition time can be optimized. Uncertainty in the estimation can be attributed to errors in the numerical fit of the spectra, which in turn can be attributed to the achieved SNR. Future studies will be aided by increases in the signal‐to‐noise ratio of the spectra, for example by using high‐sensitivity radio frequency coils^26^ and improved polarization of ^129^Xe gas,^53^ and these improvements will hopefully facilitate a time‐resolved spectroscopic imaging implementation of the global spectroscopy concept proposed here.

## 6 CONCLUSIONS

In this study, we developed a tracer kinetic model for time‐resolved NMR spectra of HP ^129^Xe in the human brain to estimate the transfer rate of HP ^129^Xe from cerebral blood to gray matter that depends on a tracer transfer constant for a known mean transit time and cerebral blood volume for gray matter. We believe this model will enable further studies to determine regional ^129^Xe tracer transfer constants with a focus of gaining insight into the pathophysiology of the BBB in diseases such as intact‐barrier edema, arterial plaque, inflammation, infarct following stroke and to aid the assessment of drug delivery to the brain. In addition, in light of the passive nature of the xenon tracer, it could serve as a cross‐reference for studies involving oxygen, water or glucose uptake, which are driven by metabolism and/or electrolytic balance.
